# PACK-CXL vs. antimicrobial therapy for bacterial, fungal, and mixed infectious keratitis: a prospective randomized phase 3 trial

**DOI:** 10.1186/s40662-021-00272-0

**Published:** 2022-01-07

**Authors:** Farhad Hafezi, Mohammed Hosny, Rohit Shetty, Boris Knyazer, Shihao Chen, Qinmei Wang, Hassan Hashemi, Emilio A. Torres-Netto, the PACK-CXL Working Group, Hanxiao Zhang, Ashraf Bora’i, Mohamed Tawfeek, Harsha Nagaraja, Sharon D’Souza, Soheila Asgari, Agha Mirsalim, Alexander Chorny, Yonit Krakauer, Bojan Pajic, Francesca Gilardoni, Nikki Hafezi, Mark Hillen, Nanji Liu, Marc-Olivier Boldi, David Tabibian, Paul R. Torgerson, Reinhard Zbinden, Hendrik Koliwer-Brandl, J. Bradley Randleman

**Affiliations:** 1 Center for Applied Biotechnology and Molecular Medicine (CABMM), University of Zurich, Zurich, Switzerland; 2 ELZA Institute, Dietikon, Switzerland; 3 Faculty of Medicine, University of Geneva, Geneva, Switzerland; 4 Department of Ophthalmology, Medical University of Wenzhou, Wenzhou, China; 5 USC Roski Eye Institute, University of Southern California Los Angeles, Los Angeles, USA; 6 Department of Ophthalmology, University of Cairo, Cairo, Egypt; 7 Narayana Nethralaya Eye Hospital and the Postgraduate Institute of Ophthalmology, Bangalore, India; 8 Department of Ophthalmology, Soroka University Medical Center, Ben-Gurion University of the Negev, Beersheba, Israel; 9 Noor Ophthalmology Research Center, Noor Eye Hospital, Tehran, Iran; 10 University Hospital Zurich, Zurich, Switzerland

**Keywords:** Photoactivated chromophore, Corneal cross-linking, Infectious keratitis, Corneal melting, PACK-CXL

## Abstract

**Background:**

Infectious keratitis is a major cause of global blindness. We tested whether standalone photoactivated chromophore corneal cross-linking (PACK-CXL) may be an effective first-line treatment in early to moderate infectious keratitis, compared with standard antimicrobial treatment.

**Methods:**

This is a randomized, controlled, multinational phase 3 clinical trial. Participants in five centers in Egypt, India, Iran, Israel, and China, aged ≥ 18 years, with infectious keratitis of presumed bacterial, fungal, or mixed origin, were randomly assigned (1:1) to PACK-CXL, or antimicrobial therapy. Outcomes measures included healing, defined as time to re-epithelialization of the corneal epithelial defect in the absence of inflammatory activity in the anterior chamber and clearance of stromal infiltrates. Treatment success was defined as the complete resolution of signs of infection.

**Results:**

Between July 21, 2016, and March 4, 2020, participants were randomly assigned to receive PACK-CXL (n = 18) or antimicrobial therapy per American Academy of Ophthalmology (AAO) guidelines (n = 21). No participants were lost to follow-up. Four eyes were excluded from the epithelialization time analysis due to treatment failure: two in the antimicrobial therapy group, and two in the PACK-CXL group. Success rates were 88.9% (16/18 patients) in the PACK-CXL group and 90.5% (19/21 patients) in the medication group. There was no significant difference in time to complete corneal re-epithelialization (*P* = 0.828) between both treatment groups.

**Conclusions:**

PACK-CXL may be an alternative to antimicrobial drugs for first-line and standalone treatment of early to moderate infectious keratitis of bacterial or fungal origin.

*Trial registration* This trial is registered at ClinicalTrials.gov, trial registration number: NCT02717871

**Supplementary Information:**

The online version contains supplementary material available at 10.1186/s40662-021-00272-0.

## Background

Severe visual impairment due to corneal infectious keratitis (corneal ulcers) represents an important cause of global blindness [1, 2]. Infectious keratitis has been described by the World Health Organization as a “silent epidemic” [3], with regions like the Indian subcontinent reporting an incidence of 800,000 new cases per year [4].

The most common pathogens are bacteria and fungi (which can also present as mixed infections), followed by *Acanthamoeba* spp. (a protozoan), and, lastly, viral keratitis (typically caused by herpes simplex virus, varicella-zoster virus, and adenovirus infection) [1]. Early intervention is crucial because there is a “window of opportunity” during early microbial invasion before the onset of ulcer formation [5]. Failure to administer timely, appropriate, and effective therapy results in poor outcomes, including total corneal necrosis, endophthalmitis, and loss of the eye [5]. Once a pathogen becomes established in the corneal stroma, the rapid growth and resulting tissue necrosis make successful medical therapy considerably more challenging, resource-intensive, and costly to treat [6]. Unfortunately, corneal scrape cultures often return negative, making a clear identification of the underlying pathogen (especially in cases of mixed infection) challenging even for experienced corneal specialists. The wrong treatment choice wastes valuable time and risks worse outcomes. Furthermore, this situation is compounded by increasing antimicrobial resistance [2]. There is clearly a growing need for a treatment modality that can effectively treat infectious keratitis in a “pathogen agnostic” manner without requiring the use of antimicrobial agents.

Corneal cross-linking (CXL) is a treatment for corneal ectasias, a family of corneal degenerative diseases that compromise the biomechanical strength of the cornea [7]. In CXL, the cornea is saturated with a chromophore (riboflavin, vitamin B_2_), which is photoactivated with 365 nm ultraviolet (UV)-A light to generate reactive oxygen species. This not only increases the biomechanical strength of the cornea through the chemical cross-linking together with collagen fibers and proteoglycans of the extracellular matrix, but also confers increased resistance to enzymatic digestion [8]. Reactive oxygen species generated by photoactivated riboflavin also kills pathogens, through cellular membrane disruption and intercalating with pathogen DNA and RNA, stopping cellular replication [9], and this effect is used in transfusion medicine to reduce the microbial load of platelet transfusions [10] and in public health interventions in developing countries to reduce the microbial load of drinking water [11]. Corneas treated with CXL are rendered sterile at the end of the procedure. In 2008, photoactivated riboflavin was first used to treat infectious keratitis, in a process termed “photoactivated chromophore for infectious keratitis corneal cross-linking” (PACK-CXL) [12].

PACK-CXL has been investigated as an adjuvant treatment to conventional antimicrobial therapy in several single-center trials for the treatment of infectious keratitis of bacterial or fungal origin, and with promising results [13–16]. Here, we compared the effect of PACK-CXL on infectious keratitis as a standalone primary treatment, with the current, standard-of-care, antimicrobial therapy.

## Methods

### Study design

**Fig. 1:**
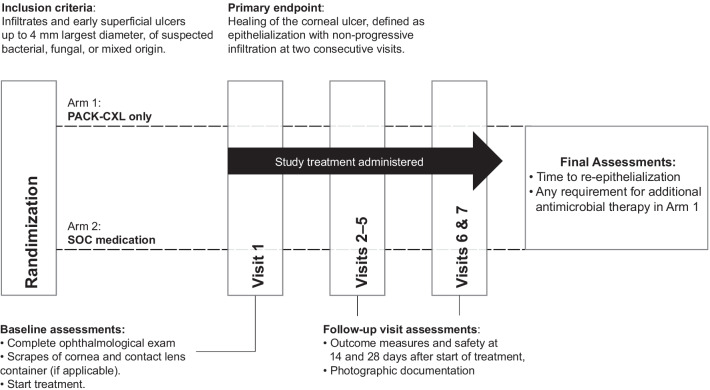
Study design schematic

This prospective, interventional, multicenter, unmasked, phase 3 randomized controlled trial was conducted at the Department of Ophthalmology at the University of Cairo (Cairo, Egypt), the Narayana Nethralaya Eye Clinic (Bangalore, India), the Department of Ophthalmology, Soroka University Medical Center (Ber Sheba, Israel), the Department of Ophthalmology Wenzhou Medical University (Wenzhou, China), and the Noor Eye Hospital (Tehran, Iran) between July 2016 and March 2020. The Department of Ophthalmology, Geneva University Hospitals, and the ELZA Institute (Dietikon/Zurich, Switzerland) served as the reading and primary investigating sites. The Institutional Review Boards of all study sites involved approved the study protocol, which adhered to all local laws and the tenets of the Declaration of Helsinki, and written informed consent was obtained by all participants before inclusion. The trial followed Good Clinical Practice for randomized controlled trials and was registered with the U.S. National Institutes of Health registry with identifier code NCT02717871 (https://clinicaltrials.gov/ct2/show/NCT02717871). Figure 1 depicts the study design.

### Participants

The study included patients aged ≥ 18 years who presented to the study sites exhibiting clinical signs of either a corneal infiltrate or an early/moderate corneal ulcer on at least one eye that was of suspected bacterial, fungal, or mixed (bacterial and fungal) origin.

Only patients with corneal infiltrates and ulcers ≤ 4 mm in diameter and showing a maximum depth of 350 μm (as assessed by either optical coherence technology (OCT) or Scheimpflug imaging) were included in the study. All lesions had to show an open epithelium with fluorescein-positive staining. All patients signed a dated informed consent form, were willing to comply with all study procedures, and make themselves available for the duration of the study.

Exclusion criteria included patients aged < 18 years, patients with clinical suspicion of non-infectious keratitis, viral or *Acanthamoeba* keratitis or sterile infiltrates, an active (or a history of) herpetic eye disease, corneal thickness < 400 µm (including the corneal epithelium), patients presenting with corneal perforation, descemetocele, systemic treatment involving steroids, immune-suppressed or immune-compromised patients, patients with diagnosed eczema or atopic dermatitis, previous keratoplasty, monocular vision, pregnant or nursing women, patients who could not be monitored with frequent clinician visits as required in the study protocol and patients who had received antimicrobial treatment less than 1 day prior to planned PACK-CXL treatment. Patients with fluorescein staining on the day of PACK-CXL treatment (but before treatment) were also excluded, as fluorescein and riboflavin have similar UV-A absorbance spectra: the presence of fluorescein would reduce the amount of UV-A energy that interacts with riboflavin, thereby reducing the antimicrobial effect of the procedure [17]. Patients who agreed to be enrolled in the study and provided informed consent were randomly assigned (1:1) by computer-generated permuted blocks stratified by center into either of the two groups: a “PACK-CXL-treated group” that received PACK-CXL treatment alone, or a “medication” (control) group that received standard-of-care medical treatment.

### Ophthalmological examination

The baseline examination (visit 1) recorded the patient’s medical history, the history of contact lens wear, the duration and type of treatment prior to the baseline visit, plus assessments of uncorrected distance visual acuity (UDVA) and corrected distance visual acuity (CDVA), slit lamp examination, determination of location and extent of the ulcer, slit lamp photography, and estimation of ulcer depth and minimal corneal thickness by OCT or Scheimpflug imaging. All pre-existing ocular treatment was interrupted at least 1 day before the treatment, after which corneal scrapes for direct smears and cultures were performed in both groups, at the first visit (day 0). Cultures were mandatory for identifying the type of pathogen, further supported by microorganism microscopy examination when needed. Conjunctival samples with swabs were not required.

After randomization and enrolment in arm 1 (PACK-CXL) or arm 2 (medication), all patients were examined as follows: visit 2 (day 1), visit 3 (day 3), visit 4 (day 5), visit 5 (typically the day of complete re-epithelialization), visit 6 (day 14, with a window of ± 2 days), visit 7 (final schedule visit at day 28, with a window of ± 2 days). If necessary, additional visits were performed at the treating physician’s discretion. In both arms, follow-up could be shortened in eyes with immediate or fast distinct improvement upon therapies. Examinations included UDVA, CDVA, slit lamp examination and photography, anterior segment OCT imaging (the specific instruments used varied by study center), assessment of the ulcer size and epithelial defect, plus an adverse event check (visits 2–7). The primary endpoint, healing, was defined as time to re-epithelialization of the corneal epithelial defect in the absence of inflammatory activity in the anterior chamber and clearance of stromal infiltrates. All adverse events were recorded.

### PACK-CXL group

Patients in the PACK-CXL group were both assessed and treated with PACK-CXL the day after the baseline visit (visit 1) to ensure no antimicrobial drugs were present in the eye. Topical anesthesia was achieved by applying oxybuprocaine and tetracaine drops every 3 min for a total of 3 times. The corneal epithelium was removed 1 mm around the borders of the infiltrate/ulcer using a hockey knife. Hypo-osmolar riboflavin solution (Ricrolin Plus^®^ 0.1% riboflavin solution, SOOFT Italia, Montegiorgio, Italy) was instilled topically on the cornea every 2 min for a total of 20 min.

The cornea was irradiated using UV-A light at 365 nm using either a UVX-2000 device (IROC Innocross, Zurich, Switzerland), KXL system (Avedro, Waltham, MA, USA), or a CCL-Vario/365 (Peschke Trade, Huenenberg, Switzerland) to deliver a total fluence of either 5.4 J/cm^2^ or 7.2 J/cm^2^, achieved by applying 9 mW/cm^2^ for either 10 min, 13 min or 20 s, respectively. Earlier cases were treated with 5.4 J/cm^2^ fluences but the protocol was modified to treat with 7.2 J/cm^2^ fluence for later cases, due to evidence that the higher fluence is more effective at killing pathogens [18]. An irradiation diameter of 8 mm centered over the ulcer was used. When the location of the ulcer demanded it, partial irradiation of the limbus was tolerated. After PACK-CXL treatment, patients were followed up until healing was complete. PACK-CXL treatment was considered a failure if a patient presented in a worse clinical situation on two consecutive follow-up controls after surgery, compared to the preoperative situation, in which case antimicrobial therapy would be initiated.

### Medication group

Cultures were performed in all patients to identify the type of pathogen present. In eyes with infectious keratitis of presumed bacterial origin, standard topical antibiotic therapy was initiated (visit 1), as recommended by the AAO Preferred Practice Pattern [19]. Antibiotics included either topical fluoroquinolones, fortified vancomycin eye drops (50 mg/mL hourly), or fortified ceftazidime eye drops (50 mg/mL hourly). In eyes with infectious keratitis of presumed fungal origin, guidelines provided by the AAO Global ONE network were used for the appropriate geographical region (usually voriconazole 1 mg/mL, but treatment options include natamycin 5%, amphotericin B 0.15–0.5%, econazole 1%, clotrimazole 1%, fluconazole 0.05%, or compounded solutions of miconazole, ketoconazole or fluconazole) [20]. In eyes with presumed mixed (bacterial and fungal origin) infections, both treatment modalities were initiated at the same time. All regimens were subject to change according to response or culture results.

### Sample size calculation and randomization

After ensuring that patients enrolled met the inclusion and had no exclusion criteria, study participants were assigned to study groups using block randomization provided by the reading center. Given the standard deviations, the sample size (n = 35) allowed an error margin of ± 8 days on the time to epithelialization difference to be achieved.

### Statistical analysis

Statistical analysis was performed with the XLSTAT Software (by Daniusoft, version 2020.1.3) and R (by R Core Team, version 4.0.2). The Mann–Whitney test was used to compare the variables between the two groups. The results were reported as mean (standard deviations) or medians and quartiles for the time to epithelialization. Cumulative distribution representations were used to illustrate both groups. A *P*-value of < 0.05 was interpreted as indicating a statistically significant difference between the two groups. In addition, the confidence interval (at 95%) on the time to epithelialization difference is reported and used to judge the sample size.

## Results

### Demographics

**Fig. 2:**
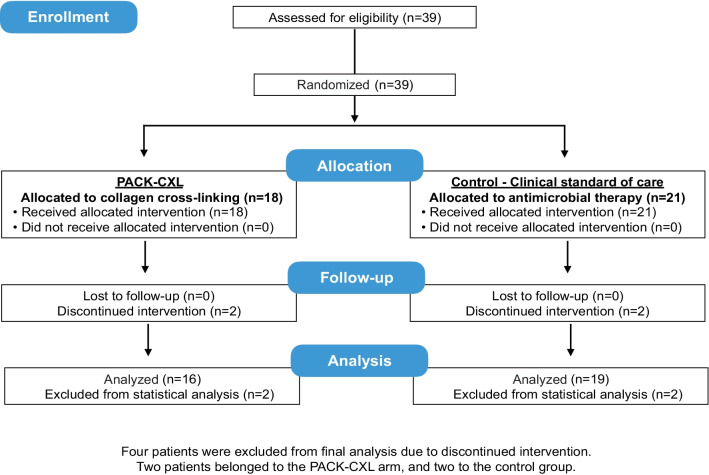
CONSORT flow chart. Four patients were excluded from the final analysis: two patients belonged to the PACK-CXL arm and needed additional antimicrobial therapy, one patient in the control group (medication only) presented with a perforation on day 7 after initiation of therapy and another patient in the control group required therapeutic keratoplasty

Between July 2016 and March 2020, when the trial was stopped after the final patient’s final follow-up visit, adults with infectious keratitis (n = 39) were assessed for eligibility and selected for inclusion in this trial and were randomized to receive either PACK-CXL alone (n = 18) or standard antimicrobial medication alone (n = 21; Figs. 1 and 2). Summary data of the baseline visit, epithelialization time and difference, and discharge visit data are described in Table 1 and in Additional file 1: Tables S1–S5.

**Table 1:** Summary of baseline visit visual acuity data and ulcer size, epithelization times in both treatment groups, and mean follow-up time and visual acuities at discharge visit

Baseline visit	Medication group (n = 21)	PACK-CXL group (n = 18)	*P*-value
	Mean (SD)	Mean (SD)	
Ulcer size (mm)	2.33 (0.87)	2.75 (1.07)	0.1564
UCVA (logMAR)	1.15 (1.03)	1.24 (0.90)	0.494
CDVA (logMAR)	0.73 (0.85)	0.76 (0.66)	0.453

Epithelization time (days)	Medication group (n = 19)	PACK-CXL group (n = 16)	*P*-value
Median	7	7	0.828
Q75%	20.5	10	
Q90%	37	19.6	
Maximum	50	28	
Mean (SD)	14.58 (14.95)	9.94 (7.18)	

Epithelization time difference between both groups			
Difference (PACK-CXL-med)	− 4.64		
Pooled SD	12.06		
Error margin	8.02		
CI (95%)	(− 12.66, 3.38)		

Discharge visit	Medication group (n = 19)	PACK-CXL group (n = 16)	*P*-value
	Mean (SD)	Mean (SD)	
Therapy/follow-up duration (days)	29.42 (12.60)	22.56 (3.82)	
UCVA (logMAR)	1.00 (1.06)	0.79 (0.68)	0.96
CDVA (logMAR)	0.54 (0.75)	0.53 (0.68)	0.96

*UCVA* uncorrected visual acuity; *CDVA* corrected distance visual acuity; *logMAR* logarithm of the minimum angle of resolution; med medication group, *PACK-CXL* photoactivated chromophore corneal cross-linking; *SD *standard deviation; *CI* confidence interval

### Baseline visit

At baseline, the groups did not differ significantly in terms of average ulcer size, which were 2.33 ± 0.87 mm and 2.75 ± 1.07 mm in the medication and PACK-CXL groups, respectively (*P* = 0.156). Likewise, the baseline UDVA and CDVA were similar in both groups. The baseline UDVA was 1.15 (Snellen: 20/283) ± 1.03 and 1.24 (20/358) ± 0.90 logMAR (*P* = 0.494), and baseline CDVA was 0.73 (20/107) ± 0.85 and 0.76 (20/115) ± 0.66 logMAR (*P* = 0.453), respectively in the medication and PACK-CXL groups.

### Etiology of microorganisms

In both groups, the microorganisms most frequently identified were Gram-positive cocci. In the PACK-CXL group (n = 18), ulcers of bacterial or fungal origin were seen in 10 (55.6%) and 4 (22.2%) eyes, respectively. In the medication group (n = 21), ulcers of bacterial or fungal origin were seen in 11 (52.4%) and 7 (33.3%) eyes. Laboratory cultures of corneal scrapes failed to grow in 4 eyes (22.2%) and 3 eyes (14.3%) in the PACK-CXL and medication groups, respectively.

### Eyes excluded from epithelialization time analysis due to treatment failures

Two patients in the PACK-CXL group and two in the medication group discontinued their assigned treatment before the end of the trial period. In the PACK-CXL group, two eyes experienced treatment failure: one had filamentous fungal keratitis and needed topical antimycotics 4 days after PACK-CXL; filaments were visualized under direct microscopy, although there was no growth at culture. The other eye showed a large increase in infiltrate size in the first 48 h after PACK-CXL was performed; *Pseudomonas aeruginosa* was identified and treated with fortified antibiotics. Two eyes in the medication group experienced treatment failure: one with *Streptococcus pneumoniae* keratitis experienced corneal perforation after 11 days of treatment and required gluing and a therapeutic contact lens. The second eye had filamentous fungal keratitis (*Fusarium* sp.) and underwent therapeutic penetrating keratoplasty due to marked corneal thinning because of delayed epithelialization.

### Time to epithelization

**Fig. 3:**
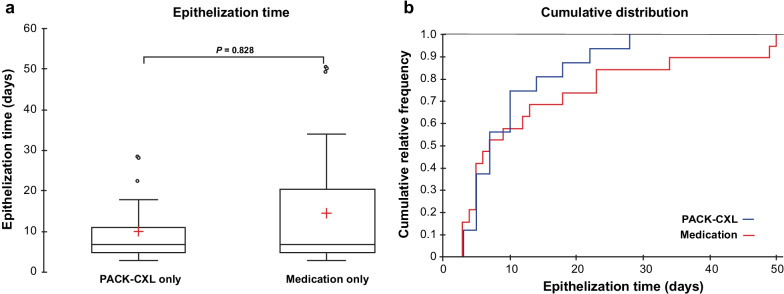
Time to epithelization. **a** Representation of the cumulative distribution of epithelial healing time in both groups. **b** Epithelialization time in both groups. Mean ± SD and median (IQR 25–75%) epithelialization time was 9.3 ± 7.1 and 7.0 (IQR 5.0–10.0) days in the PACK-CXL group (n = 16) and 14.5 ± 14.9 and 6.5 (IQR 4.7–19.2) days in the medication group (n = 19), with no significant difference between the groups (*P* = 0.824). IQR, inter-quartile range; SD, standard deviation

**Fig. 4:**
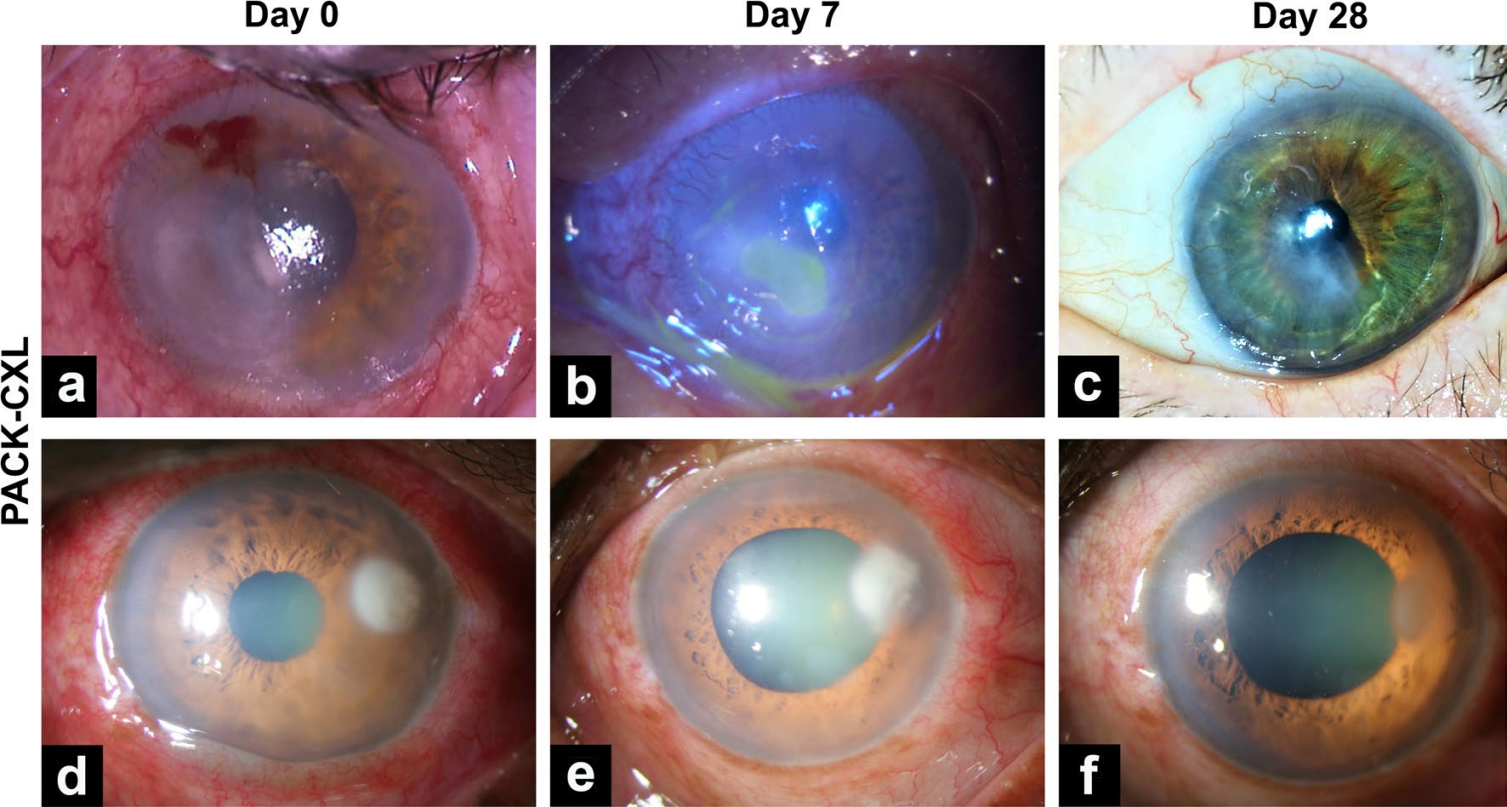
Time course of corneal ulcers in the PACK-CXL group. **a** Patient presenting with a large corneal ulcer in the left nasal inferior cornea. **b** Marked reduction of the epithelial defect and beginning reorganization at day 7 after PACK-CXL. **c** Completed epithelial closure and beginning scar formation. **d** Round and opaque ulcer in the left upper temporal cornea. **e** At day 7 after PACK-CXL, the opacity starts resolving from the temporal edge. Of note, there is a slight transient clinical worsening of the temporal lesion border due to the Jarisch–Herxheimer reaction. **f** At 28 days after PACK-CXL, infiltrate regression and full epithelial closure with beginning scar formation are noted. *PACK-CXL* photoactivated chromophore corneal cross-linking

**Fig. 5:**
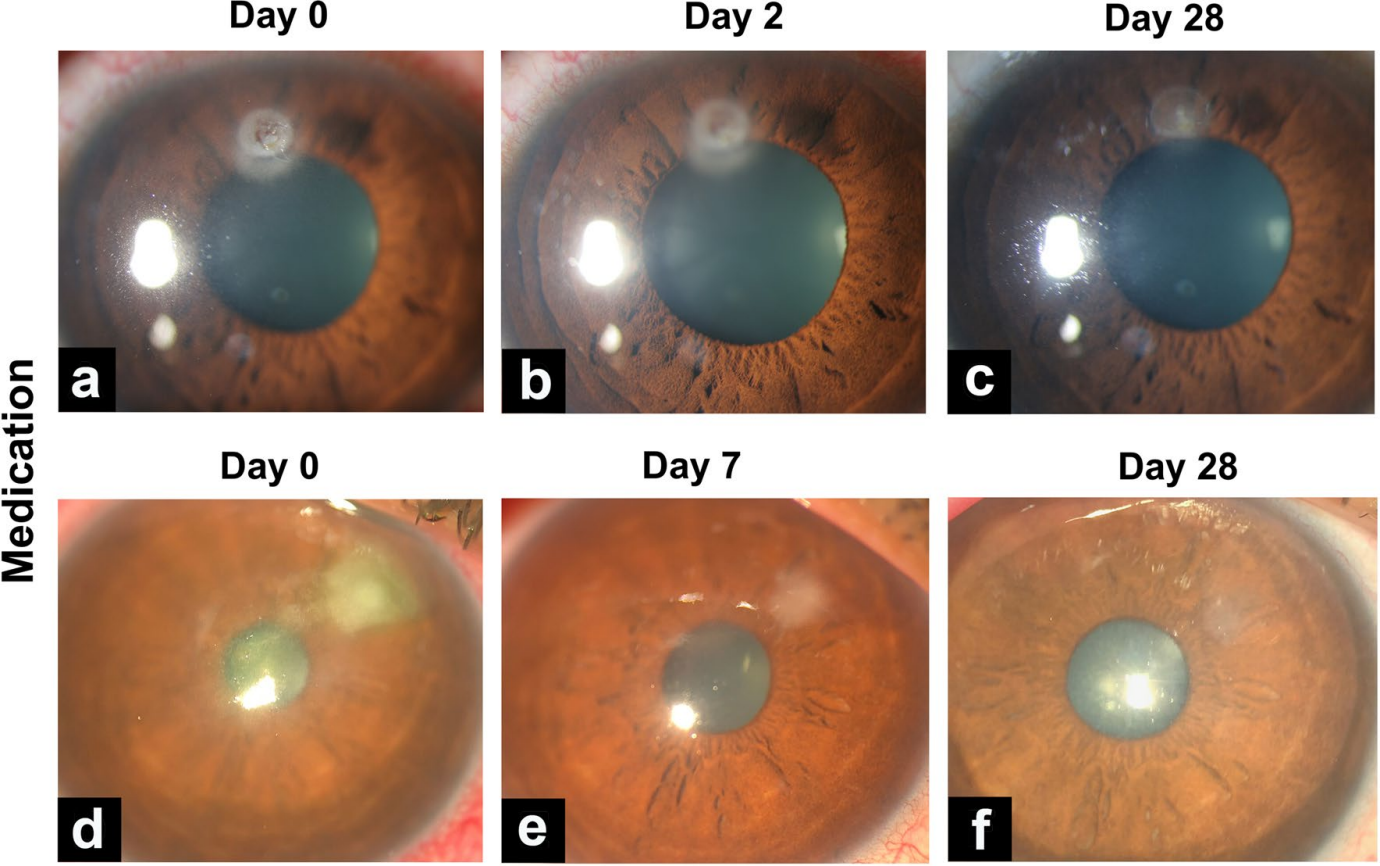
Time course of corneal ulcers in the medication group. Case 1: **a** Note the well-demarcated ulcer in middle of the upper cornea. **b** Two days later and after initiation of medication, the ulcer remains unchanged in size. **c** At day 28, complete epithelial closure and beginning scar formation are noted. Case 2: **d** Ulcer with ill-defined edges in the right upper nasal cornea. **e** 7 days later, the ulcer has markedly decreased in size. **f**. At day 28 after initiation of medication, a semi-transparent scar with full epithelial closure is noted

Four eyes that experienced treatment failure were excluded from this analysis. Epithelial healing time was calculated for both groups (n = 35), and no significant difference between groups was found (*P* = 0.828, Fig. 3a). While the median time to epithelialization was 7.0 days in both groups, the 75% and 90% quartiles were 20.5 days and 37 days in the medication group, and 10.0 and 19.6 days in the PACK-CXL group, respectively. The maximum time to epithelialization was 50 days in the standard-of-care medication-treated group, and 28 days in the PACK-CXL treated group. The epithelialization-cumulative healing time distribution is represented in Fig. 3b. The 95% confidence interval on the time difference between the two groups (PACK-CXL vs. medication) is − 12.66 to 3.38 (Table 1). No significant differences in epithelialization time were observed between eyes treated with PACK-CXL using total fluences of 5.4 or 7.2 J/cm^2^ (*P* = 0.817). Representative eyes are shown from the PACK-CXL group in Fig. 4, and the medication group in Fig. 5. In Fig. 4e, a notable point is that although there is the beginning of resolution of the infiltrate at the temporal border of the lesion, on the nasal side there seems to be a slight increase of the infiltrate, due to a Jarisch–Herxheimer reaction [21]. This phenomenon is described as an inflammatory reaction in response to endotoxin-like products released after the killing of microorganisms, which is characterized by an apparent and transient clinical worsening for the first few days.

### Visual acuity at discharge

The mean follow-up time among patients (n = 35) was 29.4 ± 12.6 days and 22.6 ± 3.8 days in the medication and PACK-CXL groups, respectively. The UDVA and CDVA at the final visit were similar in both groups: UDVA was 1.00 (20/200) ± 1.06 and 0.79 (20/123) ± 0.68 logMAR (*P* = 0.96), and CDVA was 0.54 (20/69) ± 0.75 and 0.53 (20/68) ± 0.68 logMAR (*P* = 0.96), respectively in the medication and PACK-CXL groups.

## Discussion

Infectious keratitis is a global problem, but the burden is disproportionately carried by the developing world, where those most commonly affected are middle-aged agricultural workers in their most productive years [5]. To make an impact, new treatment modalities need to help us address infectious keratitis in poorer areas of the world.

Treatment costs are high and relate to not only the medication but also follow-up visits to the treating ophthalmologist, which dominate the medication costs. In severe cases, hospitalization and round-the-clock medication administration dramatically inflate the cost of treatment. In Australia, the average cost of treating bacterial ulcers is 1400 Australian dollars, but the average cost of treating fungal ulcers is 4600 Australian dollars, owing to increased medication costs and poorer eventual visual outcomes [6, 22]. In low- to middle-income countries, many patients are unable to bear these costs and therefore cannot and do not seek treatment. Socio-economically, untreated patients often show massively impaired visual acuity, which has a direct consequence on their ability to work and provide for their families [5]. One method of reducing the costs of PACK-CXL and expanding patient access is the use of portable cross-linking devices [23]. Cross-linking, in general, tends to be performed in operating rooms, which are predominantly located in large population centers, as opposed to rural locations, which, thanks to a highly agricultural economy and a high number of corneal injuries from plant material that lead to infectious keratitis, tend to be high-need areas of infectious keratitis treatment. In this sense, a portable cross-linking device that can be used to treat patients at the slit lamp has become commercially available, and the cross-linking at the slit lamp technique has been described [23].

As a treatment for infectious keratitis, PACK-CXL holds great potential, as it can effectively kill both bacterial, fungal, and mixed bacterial/fungal keratitis, as well as rendering the cornea more resistant to enzymatic digestion—something that may help in reducing the size of the eventual scar. The first in vitro assessment of the antimicrobial efficacy of UV-A photoactivated riboflavin was published in 2008 and showed significant inhibition of bacterial growth [24]. This combination was effective against *Staphylococcus aureus*, *Staphylococcus epidermidis*, *Pseudomonas aeruginosa*, methicillin-resistant *S. aureus*, multidrug-resistant *P. aeruginosa*, and drug-resistant *S. pneumoniae*. In the same year, we reported the first clinical use of cross-linking for the treatment of infectious keratitis in five eyes resistant to antimicrobial therapy; all eyes were successfully treated using cross-linking [25]. In 2011, PACK-CXL was first used as a primary and standalone treatment for infectious keratitis in a prospective, non-randomized cohort study of 16 eyes with bacterial ulcers. The outcome was favorable, and only two eyes needed additional postoperative antibiotic treatment to heal [15].

Our understanding of how much UV energy (total fluence) can be safely delivered to the cornea has evolved. For example, it was recently observed that an increased fluence of 7.2 J/cm^2^ (as opposed to the traditional 5.4 J/cm^2^ used in CXL for ectasia treatment and initial PACK-CXL studies) can more effectively kill pathogens. The results of several single-center, non-randomized cohort studies that investigated the effects of PACK-CXL in combination with antimicrobial therapy in early, moderate, and severe cases of bacterial and fungal keratitis were published between 2013 and 2017. Overall, these showed that PACK-CXL that delivered a total of 7.2 J/cm^2^ fluence, is more effective in killing bacteria than fungi [26, 27], and that the outcomes were more favorable in small ulcers (≤ 4 mm in diameter) than in mid-sized and large ulcers (> 4 mm in diameter), irrespective of the pathogen [16, 27, 28]. The study presented here commenced in 2016 and initially used UV irradiation protocols that delivered 5.4 J/cm^2^ fluence, however, the evidence from these smaller studies prompted the switch to the delivery of 7.2 J/cm^2^ fluence, although the study was not sufficiently powered to detect any difference in efficacy between the two fluences.

The study presented here shows that both PACK-CXL and standard-of-care medication result in similar epithelial healing rates. Major complication rates were also similar in both treatment arms. Given the standard deviations observed, the sample size (n = 35) enabled the calculation of an error margin of ± 8 days on the difference in time to epithelialization between both treatment groups. Bearing in mind that the median time to epithelialization in both groups was 7 days, and if we take a pessimistic scenario of 8 days additional epithelialization time with PACK-CXL, this might translate to additional days of patient discomfort, but clinically, this is acceptable as a positive outcome is still achieved, and issues with antibiotic resistance and medication requirements are avoided. Nevertheless, our study shows re-epithelialization times comparable to those achieved with PACK-CXL reported previously [14]. It is worth noting that a small (~ 1 mm) debridement of the corneal epithelium around the infection site is made to allow better riboflavin penetration into the ulcer. However, we do not believe that this debridement results in any increase in the risk of post-surgical infection. This region receives the same pathogen-killing effects of PACK-CXL as the adjacent region of the ulcer, and just like any surgery that involves some measure of epithelial debridement, any risk of subsequent corneal infection is therefore related to post-procedural handling of the open corneal surface. Furthermore, and most importantly, the infection potentially expands into the cornea even into areas where infiltrates are not yet clinically or distinctly visible biomicroscopically, so here again a margin widening would potentially bring advantages in eliminating microorganisms in such regions that are not yet clinically distinguishable.

When viewed from the perspective of an ophthalmologist in a developing country, PACK-CXL as a sole treatment is equally successful as medication in treating keratitis, and in most cases, PACK-CXL does so in a single, short treatment session, which is of great importance when patients may only be able to afford a single doctor visit. Standard-of-care therapy usually requires considerably more intensive intervention by medical staff, and often, the cost burdens can be prohibitive to the patients in these regions. When viewed from the perspective of an ophthalmologist in a developed country, PACK-CXL may help to reduce the size of the resulting scar by increasing the stroma’s resistance to enzymatic digestion, an effect that cannot be provided by antimicrobial medication.

Although visual acuity was measured, it was not specified as an efficacy endpoint, as both the size and location of the ulcer and the scar that results from it can make a considerable difference to a patient’s vision (a small ulcer/scar in the center of the cornea will degrade vision more than a larger ulcer/scar on the periphery of the cornea).

The overall success rate for first-line standalone PACK-CXL in this study was 88.9% (16/18). This success rate was achieved using a total UV-A radiant exposure (fluence) that respected the limits for endothelial cell safety that were validated at the time of the study [29]. A recent study using multiphoton tomography shows that the human endothelium can support distinctly higher fluences than previously assumed [30]. This is in line with clinical studies on customized CXL where fluences up to 15.0 J/cm^2^ were used without inducing endothelial damage [31]. Increasing fluence translates to increased corneal tissue levels of reactive oxygen species, potentially further increasing the killing rate of bacteria and fungi. This was confirmed recently by our group when high-fluence PACK-CXL was shown to increase antibacterial efficacy in six distinct bacterial strains [18].

Several studies have evaluated the effect of PACK-CXL as an adjunct therapy in the treatment of infectious keratitis of bacterial and fungal origin. These studies showed that the addition of PACK-CXL was able to significantly accelerate the time to healing [14, 32]. Here, we evaluated PACK-CXL as a first-line standalone treatment in a prospective randomized controlled trial and report healing rates similar to antimicrobial therapy. Based on our recent laboratory data [18], the fluences used in our clinical study (5.4 or 7.2 J/cm^2^) can be increased, potentially further improving clinical outcomes. PACK-CXL is therefore attractive as an adjunct to standard-of-care treatment (it should accelerate healing time and may reduce the final scar size) but can be used, if circumstances dictate, as a standalone therapy, in the knowledge that it has similar efficacy to standard-of-care antimicrobial treatment, albeit with a potentially slower time to epithelial closure.

This study had a number of limitations. The small cohort size meant that only a difference in epithelialization times of 8 days or more could be detected. Also, the sample size was too small to determine if there was a difference in PACK-CXL efficacy in treating ulcers of bacterial, fungal, or mixed origin, or whether the higher pathogen killing effect of 7.2 J/cm^2^ fluence was clinically distinguishable from 5.4 J/cm^2^ fluence. Moreover, not all corneal scrapes resulted in positive cultures, which is a common occurrence in clinical practice, but meant that we did not have a complete picture of the pathogens present in every case. The difference between the quartiles of both groups in the time to epithelialization shown in Fig. 3 is interesting. The greater variation in the medication group could be attributed to a slightly higher prevalence of fungal keratitis in the medication group (33%) than in the PACK-CXL group (22%), as the epithelialization time for fungal keratitis is potentially longer. On the other hand, this limitation cannot be controlled given the randomized nature of the study. Therefore, the possible bias here represented by the discrete etiological variation between groups is potentially smaller than that of a non-randomized study would be. Finally, the small sample size was a function of the strict data collection requirements of the trial and the requirement of experienced cornea specialists to reliably classify ulcer types on patient presentation. Inclusion of smaller rural sites would have increased the number of eyes with ulcers requiring treatment, but the lack of data quality management systems that the larger centers had would have limited our confidence in the results achieved.

## Conclusion

PACK-CXL has already been shown to reduce the healing time and final scar size when used in combination with standard-of-care antimicrobial therapy, but this study shows that PACK-CXL represents a viable standalone and potentially first-line alternative to antibiotics for the treatment of small bacterial and fungal corneal ulcers. PACK-CXL can help counter increasing antimicrobial resistance, and as the technique evolves and becomes more established, it may change the dogma that antimicrobial therapy is always first-line therapy and move antimicrobial agents from a current standard-of-care role to a secondary, supporting role instead.

### Supplementary Information

**Additional file 1: Table S1. **Pathogen type, treatment administered, and uncorrected and correct distance visual acuity (CDVA and UDVA) scores in logMAR. **Table S2.** Details of the sizes of ulcers and epithelial defects in the Swiss PACK-CXL study. **Table S3.** Details anterior chamber findings in the Swiss PACK-CXL study. **Table S4.** Details of corneal thickness (µm) measurements, where available. **Table S5.** Epithelization time and study follow-up duration.

## Data Availability

The datasets used and/or analyzed during the current study are available from the corresponding author upon reasonable request.

## Appendix

PACK-CXL Working Group

**Table:**

China	Hanxiao Zhang, MD (Department of Ophthalmology, Wenzhou Medical University, Wenzhou)
Egypt	Ashraf Bora'i, MD; Mohamed Tawfeek, MD (Department of Ophthalmology, Zagazig University, Zagazig)
India	Harsha Nagaraja, MD; Sharon D’Souza, MD (Narayana Nethralaya Hospital, Bangalore)
Iran	Soheila Asgari, Ph.D.; Agha Mirsalim, MD (Noor Ophthalmology Research Center, Noor Eye Hospital, Tehran)
Israel	Alexander Chorny, MD; Yonit Krakauer, MD (Department of Ophthalmology, Soroka University Medical Center, Ben-Gurion University of the Negev, Beer-Sheva)
Serbia	Bojan Pajic, MD, Ph.D. (Faculty of Sciences, Department of Physics, University of Novi Sad, Novi Sad)
Switzerland	Francesca Gilardoni, MD; Nikki Hafezi, MAS IP ETHZ; Mark Hillen, Ph.D., Nanji Liu, MD (ELZA Institute, Dietikon). Marc-Olivier Boldi, Ph.D. (Department of Operations, University of Lausanne, Lausanne). David Tabibian, MD (Department of Ophthalmology, University of Lausanne, Lausanne). Paul R. Torgerson, Ph.D., VetMB, DipECVPH (Department of Biostatistics, University of Zurich, Zurich). Reinhard Zbinden, MD, Ph.D.; Hendrik Koliwer-Brandl, Ph.D. (Institute of Medical Microbiology, University of Zurich, Zurich)
USA	J. Bradley Randleman, MD (Cleveland Clinic Foundation, Cole Eye Institute, Cleveland, OH, USA)
