# Supplementary material for: PACK-CXL vs. antimicrobial therapy for bacterial, fungal, and mixed infectious keratitis: a prospective randomized phase 3 trial
Source: Eye Vis (Lond). 2022 Jan 7;9:2. doi: 10.1186/s40662-021-00272-0 (PMC8742313; doi:10.1186/s40662-021-00272-0)
Supplement: Supplementary file 1 — Additional file 1: Table S1. Pathogen type, treatment administered, and uncorrected and correct distance visual acuity (CDVA and UDVA) scores in logMAR. Table S2. Details of the sizes of ulcers and epithelial defects in the Swiss PACK-CXL study. Table S3. Details anterior chamber findings in the Swiss PACK-CXL study. Table S4. Details of corneal thickness (µm) measurements, where available. Table S5. Epithelization time and study follow-up duration. [file 40662_2021_272_MOESM1_ESM.docx]

**Table S1**. Pathogen type, treatment administered, and uncorrected and correct distance visual acuity (CDVA and UDVA) scores in logMAR.

| No. | Age  (years) | Type of pathogen | Treatment | Day 0 | | Day 1 | | Day 3 | | Day 5 | | Day 7 | | Day 14 | | Day 28 | |
| --- | --- | --- | --- | --- | --- | --- | --- | --- | --- | --- | --- | --- | --- | --- | --- | --- | --- |
|  |  |  |  | UDVA | CDVA | UDVA | CDVA | UDVA | CDVA | UDVA | CDVA | UDVA | CDVA | UDVA | CDVA | UDVA | CDVA |
| 1 | 35 | *Aspergillus* sp. | Medication | 0.50 | 0.30 | 0.50 | 0.40 | 0.80 | 0.60 | - | - | - | - | 0.70 | 0.50 | 0.40 | 0.20 |
| 2 | 25 | *Staphylococcus aureus* | Medication | 2.00 | 0.00 | 2.00 | 0.00 | 2.00 | 0.00 | - | - | - | - | 2.00 | 0.00 | 2.00 | 0.00 |
| 3 | 32 | *Staphylococcus aureus* | PACK-CXL 5.4 J/cm² | 0.80 | 0.20 | 0.80 | 0.20 | 0.80 | 0.00 | 0.80 | 0.00 | - | - | 0.80 | 0.00 | 0.80 | 0.00 |
| 4 | 55 | *Aspergillus niger* | Medication | 0.10 | 0.10 | 0.10 | 0.10 | 0.10 | 0.10 | 0.10 | 0.10 | 0.10 | 0.10 | 0.10 | 0.10 | 0.10 | 0.10 |
| 5 | 21 | N/A | Medication | 0.20 | 0.20 | 0.20 | 0.00 | 0.10 | 0.00 | - | - | - | - | 0.00 | 0.00 | 0.00 | 0.00 |
| 6 | 29 | *Aspergillus flavus* | Medication | 0.60 | 0.40 | 0.80 | 0.60 | 0.90 | 0.70 | - | - | - | - | 0.10 | 0.00 | 0.10 | 0.00 |
| 7 | 51 | *Staphylococcus aureus* | PACK-CXL 5.4 J/cm² | 2.00 | 1.00 | 2.00 | 2.00 | 2.00 | 2.00 | 2.00 | 2.00 | 2.00 | 1.00 | 2.00 | 1.00 | 2.00 | 1.00 |
| 8 | 32 | *Staphylococcus aureus* | Medication | 0.00 | 0.00 | 0.00 | 0.00 | 0.00 | 0.00 | - | - | - | - | 0.00 | 0.00 | 0.00 | 0.00 |
| 9 | 53 | *Staphylococcus aureus* | PACK-CXL 5.4 J/cm² | 0.60 | 0.60 | 0.80 | 0.70 | 0.70 | 0.70 | 0.70 | 0.60 | 0.60 | 0.60 | 0.60 | 0.60 | 0.60 | 0.60 |
| 0 | 19 | *Pseudomonas aeruginosa* | PACK-CXL 5.4 J/cm² | 2.00 | 0.80 | 0.80 | 0.40 | 0.80 | 0.20 | - | - | - | - | 0.60 | 0.10 | 0.60 | 0.00 |
| 11 | 46 | *Klebsiella* sp.*, Serratia marcescens, Morganella morganii, Escherichia coli* | PACK-CXL 5.4 J/cm² | 0.80 | 0.20 | 0.80 | 0.10 | 0.80 | 0.00 | - | - | - | - | 0.20 | 0.00 | 0.00 | 0.00 |
| 12 | 41 | No growth | Medication | 0.20 | 0.00 | 0.90 | - | 0.00 | 0.00 | 0.00 | 0.00 | - | - | - | - | 0.00 | 0.00 |
| 13 | 54 | *Staphylococcus aureus* | Medication | 0.10 | 0.10 | 0.10 | 0.10 | 0.10 | 0.10 | - | - | - | - | 0.10 | 0.10 | 0.10 | 0.10 |
| 14 | 38 | *Streptococcus pneumoniae* | Medication | 0.00 | 0.00 | 0.10 | 0.10 | 0.20 | 0.20 | 0.60 | 0.30 | 2.00 | 2.00 | - | - | 2.00 | 2.00 |
| 15 | 33 | *Staphylococcus aureus* | Medication | 0.00 | 0.00 | 0.00 | 0.00 | 0.00 | 0.00 | - | - | - | - | 0.00 | 0.00 | 0.00 | 0.00 |
| 16 | 76 | No growth | Medication | 0.20 | 0.10 | 0.20 | 0.10 | 0.20 | 0.10 | 0.10 | 0.00 | - | - | 0.10 | 0.00 | 0.10 | 0.00 |
| 17 | 43 | N/A | PACK-CXL 7.2 J/cm² | 0.30 | 0.40 | 0.20 | 0.50 | 0.50 | 0.60 | 0.50 | 0.50 | 0.60 | 0.60 | 0.80 | 0.80 | 0.60 | 0.90 |
| 18 | 56 | *Aspergillus* sp. | PACK-CXL 7.2 J/cm² | 0.10 | 0.00 | 0.30 | 0.20 | 0.20 | 0.00 | - | - | 0.10 | 0.00 | 0.10 | 0.00 | 0.10 | 0.00 |
| 19 | 71 | N/A | PACK-CXL 7.2 J/cm² | 0.50 | 0.50 | 0.10 | 0.30 | 0.20 | 0.20 | - | - | - | - | - | - | 0.70 | 0.70 |
| 20 | 29 | *Pseudomonas aeruginosa* | PACK-CXL 5.4 J/cm² | 1.00 | 1.00 | 3.00 | 3.00 | 3.00 | 3.00 | 3.00 | 3.00 | 3.00 | 3.00 | 3.00 | 3.00 | 3.00 | 3.00 |
| 21 | 23 | *Staphylococcus aureus* | Medication | 2.00 | 0.80 | 2.00 | 0.80 | 2.00 | 0.70 | - | - | - | - | 2.00 | 0.70 | 2.00 | 0.70 |
| 22 | 23 | N/A | Medication | 2.00 | 0.50 | 2.00 | 0.50 | 2.00 | 0.30 | 2.00 | 0.20 | - | - | 2.00 | 0.20 | 2.00 | 0.20 |
| 23 | 87 | N/A | PACK-CXL 7.2 J/cm² | 2.00 | 2.00 | 2.00 | 2.00 | 2.00 | 2.00 | 2.00 | 2.00 | - | - | 1.00 | 1.00 | 0.90 | 0.80 |
| 24 | 35 | No growth | PACK-CXL 7.2 J/cm² | 0.00 | 0.00 | 0.18 | 0.00 | 0.18 | 0.00 | 0.18 | 0.00 | 0.18 | 0.00 | 0.00 | 0.00 | 0.00 | 0.00 |
| 25 | 19 | No growth | Medication | 2.00 | 0.90 | 1.10 | 1.00 | 1.10 | 0.70 | 0.80 | 0.60 | 0.80 | 0.60 | 0.80 | 0.30 | 0.80 | 0.20 |
| 26 | 77 | No growth | PACK-CXL 7.2 J/cm² | 0.60 | 0.50 | 0.76 | 0.60 | 0.60 | 0.48 | 0.6 | 0.48 | 0.48 | 0.40 | 0.40 | 0.30 | 0.30 | 0.20 |
| 27 | 18 | No growth | PACK-CXL 7.2 J/cm² | 2.00 | 1.00 | 1.00 | 0.80 | 0.80 | 0.80 | 0.80 | 0.80 | - | - | - | - | 0.70 | 0.70 |
| 28 | 19 | *Pseudomonas aeruginosa* | PACK-CXL 7.2 J/cm² | 0.70 | 0.30 | 0.80 | 0.48 | 0.60 | 0.48 | 0.48 | 0.40 | 0.48 | 0.30 | 0.48 | 0.00 | 0.50 | 0.00 |
| 29 | 84 | *Staphylococcus lugdunensis, Corynebacterium* sp. | Medication | 1.00 | 0.80 | 1.00 | 0.90 | 0.90 | 0.80 | 0.80 | 0.80 | 0.80 | 0.60 | 0.60 | 0.48 | 0.50 | 0.40 |
| 30 | 43 | *Cladosporium* spp. | Medication | 2.00 | 2.00 | - | - | - | - | - | - | - | - | - | - | 3.00 | 2.00 |
| 31 | 69 | *Staphylococcus aureus* | Medication | 3.00 | 2.00 | - | - | - | - | - | - | - | - | - | - | 2.00 | 2.00 |
| 32 | 63 | *Fusarium* sp. | Medication | 2.00 | 1.60 | - | - | - | - | - | - | - | - | - | - | 1.50 | 1.60 |
| 33 | 54 | *Penicillium* sp. | Medication | 3.00 | 3.00 | - | - | - | - | - | - | - | - | - | - | 3.00 | 2.00 |
| 34 | 19 | *Fusarium* sp. | Medication | 1.60 | 1.30 | - | - | - | - | - | - | - | - | - | - | 1.60 | 1.30 |
| 35 | 38 | *Candida albicans* | Medication | 1.60 | 1.30 | - | - | - | - | - | - | - | - | - | - | 1.30 | 1.00 |
| 36 | 62 | *Candida albicans* | PACK-CXL 5.4 J/cm² | 3.00 | 2.00 | - | - | 3.00 | 2.00 | 3.00 | 2.00 | 3.00 | 2.00 | 3.00 | 2.00 | 2.00 | 2.00 |
| 37 | 29 | *Aspergillus fumigatus* | PACK-CXL 5.4 J/cm² | 1.00 | 0.30 | - | - | 1.00 | 0.48 | 1.00 | 0.48 | 1.00 | 0.30 | 1.00 | 0.30 | 1.00 | 0.20 |
| 38 | 39 | *(atypical) mycobacteria* | PACK-CXL 5.4 J/cm² | 1.60 | 0.90 | 2.00 | 2.00 | 1.60 | 1.60 | 1.60 | 1.60 | 1.60 | 1.60 | 1.60 | 1.00 | 1.00 | 0.90 |
| 39 | 40 | N/A | PACK-CXL 5.4 J/cm² | 3.00 | 2.00 | 3.00 | 3.00 | 3.00 | 3.00 | 3.00 | 3.00 | 3.00 | 3.00 | 2.00 | 2.00 | 2.00 | 2.00 |

**Table S2**. Details of the sizes of ulcers and epithelial defects in the Swiss PACK-CXL study.

| No. | Day 0 | Day 1 | Day 3 | Day 5 | Day 7 | Day 14 | Day 28 |
| --- | --- | --- | --- | --- | --- | --- | --- |
|  | **Ulcer and epithelial defect (mm)** | | | | | | |
| 1 | 2.0×1.5; 1.5 | 2.0×1.5; 3.0 | 4×4; 4 | 4.0×3.5; 3.0 | 3.5×3.5; 2.0 | 3.0×2.0; 2 | 0 |
| 2 | 1.0×1.0; 1.0 | 1.0×1.0; 0.5 | 0.5×0.5; healed | - | - | 0 | 0 |
| 3 | 1.0×1.0; 1.0 | 1.0×1.0; 1.5 | 0.5×0.5; 0.5 | 0.5×0.2; healed | 0.5×0.2; 0.1 | 0 | 0 |
| 4 | 2.0×2.0; 2.0 | 2.0×2.0; 1.5 | 1.5×1.5; 1 | 1.0×1.5; 0.2 | 0.5×0.5; 0 | 0.3×0.3; 0 | 0 |
| 5 | 1.5×1.5; 1.0 | 1.5×1.5; 0.5 | 1× 1; healed | - | - | 0 | - |
| 6 | 0.5×1.0; erosion | 1.5×1.5; erosion | 1.5×1.5; erosion | 0.5×1.0; erosion | 0.5×0.5; erosion | 0.5×0.5; closed | 0.5×0.3; closed |
| 7 | 2.0×2.0; 3.0 | 2.0×2.0; 4.0 | 2×2; 2 | 2.0×2.0; 1.0 | 2.0×1.0; 0.5 | 1.0×1.0; 0 | 0 |
| 8 | 2.0×2.0; 2.0 | 2.0×2.0; 1.0 | 1×1; 0 | - | - | 0 | 0 |
| 9 | 2.0×2.0; 3.0 | 3.0×3.0; 4.0 | 3×2; 2 | 2.0×2.0; 1.0 | 2.0×1.0; 0.5 | 1.0×1.0; 0 | 0 |
| 10 | 1.5×1.5; erosion 2.0 | 1.2×1.2; erosion 0.5 | 0.5×0.5; 0.2 | 0.3×0.3; closed | - | 0 | 0 |
| 11 | 0.5×0.5; 2.0 | 0.2×0.2; 0.2 | 0.1×0.1; closed | - | - | 0 | 0 |
| 12 | 2.0×2.0; erosion 1.0 | - | 1×0.5; breakdown 0.2 | closed | - | - | - |
| 13 | 2.0×2.0; 2.0 | 2.0×2.0; 1.0 | 1.5×1; 0 | - | - | 0 | 0 |
| 14 | 2.0×2.0; 2.0 | 2.0×2.0; 4.0 | 4×3; 4 | 4.0×4.0;4.0 | 4.0×4.0; 4.0 |  |  |
| 15 | 2.0×2.0; 2.0 | 2.0×2.0; 1.0 | 2×1.5; 0 | - | - | 0 | 0 |
| 16 | 1.5×1.0; breakdown | 1.5×1.0; breakdown 1.0×1.0 | 2×1; breakdown 0.2 | closed | - | closed | closed |
| 17 | 2.0×2.0; breakdown | 0; 3.0 | 0; 3 | 0; 3.0 | 0; 3.0 | 0; 3.0 | 0; 3.0 |
| 18 | 2.0×2.0; 2.0 | 2.0×2.0; 4.0 | 2.0×2.0; 2.0 | 2.0×2.0; 0.5 | 1.0×1.5; 0.1 | 0.5×0.5; 0 | 0 |
| 19 | 3.0×3.0; breakdown | 0; 3.0 | 2.0×2.0; 3.0 | - | - | - | - |
| 20 | 2.0×2.0; breakdown | 4×7; breakdown | 4.0×7.0; breakdown | 4.0×7.0; breakdown | 4.0×7.0; breakdown | 4.0×7.0; breakdown | - |
| 21 | 2.0×2.0; 2.0 | 2×2; 3 | 1.0×1.0; healed | - | - | 0 | - |
| 22 | 1.0×3.0; 3.0 | 3×3; 4 | 3.0×2.0; 2.0 | 2.0×2.0; 0 | - | 0 | 0 |
| 23 | 3.0×4.0; breakdown | 3.0×4.0; Yes | 3.0×3.0; 4.0×4.0 | 3.0×2.5; 3.0×3.0 | 2.0×1.5; 2.0×2.0 | 1.2×1.0; 1.0×1.0 | 0 |
| 24 | 1.5×1.5; 0.5 | 3.0×3.0; 0.5 | 1.5×1.5 | 1.0×1.0 | 0 | 0 | 0 |
| 25 | 2.5×2.5 | 2.5×2.5 | 2.5×2.5 | 1.0×1.0 | 0.5×0.5 | 0 | 0 |
| 26 | 2.5×2.5 | 3.5×3.5 | 2.5×2.5 | 1.5×1.5 | 1.0×1.0 | 0 | 0 |
| 27 | 0.5×0.5 | 0.3×0.3 | 0 | 0 | - | - | - |
| 28 | 2.5×2.5 | 3.5×3.5 | 2.5×2.5 | 1.5×1.5 | 1.0×1.0 | - | - |
| 29 | 2.5×2.0 | 3.0×2.0 | 2.5×1.5 | 1.3×1.0 | 0.5×0.5 | 0 | 0 |
| 30 | 1.0×1.0 | - | - | - | - | - | - |
| 31 | 2.0×1.0 | - | - | - | - | - | - |
| 32 | 3.0×3.0 | 3.0 | 3.0 | 4.0 | 4.0 | 4.0 | 4.0 |
| 33 | 4.0×4.0 | - | - | 3.0×3.0 | - | - | - |
| 34 | 2.0×2.0 | - | - | - | - | 2.0; 2.0 | 1.0; 1.0 |
| 35 | 2.0×2.0 | - | - | - | - | 1.0; 1.0 | - |
| 36 | 2.0×2.0 | - | - | - | 1.0×1.0 | 0 | - |
| 37 | 2.0×2.0 | - | 1.0×1.0 | - | 0 | - | - |
| 38 | 2.0×2.0 | - | - | 2.0×1.0 | 1.0×1.0 | 0 | - |
| 39 | 3.0×2.0 | - | - | 1.0×1.0 | 0 | - | - |

**Table S3**. Details anterior chamber findings in the Swiss PACK-CXL study.

| No. | Day 0 | Day 1 | Day 3 | Day 5 | Day 7 | Day 14 | Day 28 |
| --- | --- | --- | --- | --- | --- | --- | --- |
|  | **Anterior chamber findings** | | | | | | |
| 1 | None | None | Cell + Flare ++ | Cell ++ Flare +++ | Cell + Flare ++ | Cell + Flare + | None |
| 2 | None | None | None | None | None | None | None |
| 3 | None | None | None | None | None | None | None |
| 4 | Cell + Flare + | Cell + Flare + | Cell +  Flare ++ | Flare + | None | None | None |
| 5 | Cell ++ Flare ++ | Cell+  Flare + | None | None | None | None | None |
| 6 | None | None | Cell +  Flare ++ | Flare + | None | None | None |
| 7 | Cell +++ Flare +++ | Cell ++++ Flare ++++ | Cell ++++ Flare ++++ | Cell +++ Flare ++++ | Flare + | None | None |
| 8 | None | None | None | None | None | None | None |
| 9 | None | None | None | None | None | None | None |
| 10 | Cell ++ Flare ++ | Cell ++ Flare ++ | Cell + Flare + | None | None | None | None |
| 11 | None | None | None | None | None | None | None |
| 12 | None | None | None | None | None | None | None |
| 13 | None | None | None | None | None | None | None |
| 14 | None | None | None | None | None |  |  |
| 15 | None | None | None | None | None | None | None |
| 16 | None | None | None | None | None | None | None |
| 17 | None | None | None | None | None | None | None |
| 18 | None | None | None | None | None | None | None |
| 19 | None | None | None | None | - | - | - |
| 20 | Cell ++ Flare + | Cell +++ Flare ++ | Cell +++ Flare ++ | Cell +++ Flare ++ | Cell +++ Flare ++ | Cell +++ Flare ++ | None |
| 21 | None | None | None | None | None | None | None |
| 22 | None | None | None | None | None | None | None |
| 23 | None | Cell +++ Flare +++ | Cell ++ Flare ++ | Cell + Flare ++ | Cell + Flare + | None | None |
| 24 | None | None | None | None | None | None | None |
| 25 | None | None | None | None | None | None | None |
| 26 | Cell ++  Flare ++ | Flare + | None | None | None | None | None |
| 27 | Flare + | None | None | None | - | - | - |
| 28 | Cell +++  Flare ++ | Cell ++ Flare + | Cell + | None | - | - | - |
| 29 | Cell ++  Flare ++ | Cell ++ Flare ++ | Cell ++ Flare ++ | Flare + | None | None | None |
| 30 | None | - | - | - | - | - | - |
| 31 | Cell + | - | - | - | - | - | - |
| 32 | Cell +  Flare +  hypopyon 3 mm | Cell + Flare + Hypopyon + | Cell + Flare + Hypopyon 4 mm | Cell + Flare + Hypopyon + | Cell + Flare + Hypopyon + | Cell + Flare + Hypopyon + | Cell + Flare + Hypopyon + |
| 33 | Cell +  Flare + | - | - | None | - | - | - |
| 34 | Cell +  Flare +  hypopyon 2 mm | - | - | - | - | None | - |
| 35 | None | - | - | - | - | None | - |
| 36 | Cell +  Flare + | Cell + Flare + Hypopyon 2 mm | Hypopyon 1 mm | None | None | - | - |
| 37 | None | - | None | - | None | - | - |
| 38 | None | - | - | None | None | None | - |
| 39 | Cell +  Flare +  Hypopyon 4 mm | - | Hypopyon 2 mm | - | Hypopyon 1 mm | None | - |

**Table S4**. Details of corneal thickness (µm) measurements, where available.

| No. | Day 0 | Day 1 | Day 3 | Day 5 | Day 7 | Day 14 | Day 28 |
| --- | --- | --- | --- | --- | --- | --- | --- |
|  | **Corneal thickness (µm)** | | | | | | |
| 1 | 513 | 543 | 544 | 533 | 516 | 541 | 548 |
| 2 | 533 | 528 | 521 | - | - | 528 | 523 |
| 3 | 548 | 516 | 532 | 497 | 546 | 533 | 514 |
| 4 | 516 | 522 | 528 | 534 | 511 | 532 | 542 |
| 5 | 556 | 532 | 498 | - | - | 563 | - |
| 6 | 563 | - | - | - | - | - | 536 |
| 7 | 564 | 569 | 553 | 562 | 548 | 544 | 526 |
| 8 | 532 | 528 | 538 | - | - | 528 | 542 |
| 9 | 544 | 531 | 539 | 541 | 522 | 542 | 547 |
| 10 | 686 | 684 | 676 | 684 | - | 764 | 630 |
| 11 | 512 | 512 | 512 | - | - | - | 520 |
| 12 | 527 | - | 527 | 527 | - | - | - |
| 13 | 573 | 568 | 572 |  | - | 561 | 565 |
| 14 | 538 | 544 | 534 | 551 | - | - | - |
| 15 | 528 | 531 | 518 | - | - | 531 | 514 |
| 16 | 558 | 535 | 530 | 535 | - | 538 | 520 |
| 17 | 645 | 613 | 560 | 547 | 539 | 541 | 547 |
| 18 | 516 | 532 | 522 | 533 | 531 | 522 | 536 |
| 19 | 543 | 395 | 602 | - | - | - | - |
| 20 | - | - | - | - | - | - | - |
| 21 | 543 | 533 | 526 | - | - | 533 | - |
| 22 | 522 | 511 | 519 | - | - | 525 | 522 |
| 23 | 1105 | 1008 | - | - | - | - | 584 |
| 24 | 491 | - | - | - | - | - |  |
| 25 | - | - | - | - | - | - | 414 |
| 26 | 410 | - | - | - | - | - | 517 |
| 27 | 547 | - | - | 487 | - | - | - |
| 28 | 551 | - | - | - | 586 | - | - |
| 29 | 700 | - | - | - | - | 633 | - |
| 30 | - | - | - | - | - | - | - |
| 31 | - | - | - | - | - | - | - |
| 32 | - | - | - | - | - | - | - |
| 33 | - | - | - | - | - | - | - |
| 34 | - | - | - | - | - | - | - |
| 35 | - | - | - | - | - | - | - |
| 36 | - | - | - | - | - | - | - |
| 37 | - | - | - | - | - | - | - |
| 38 | - | - | - | - | - | - | - |
| 39 | - | - | - | - | - | - | - |

**Table S5.** Epithelization time and study follow-up duration.

| No | Epithelization completed (days) | Study follow-up (days) | Excluded from analysis |
| --- | --- | --- | --- |
| 1 | 23 | 28 | No |
| 2 | 3 | 28 | No |
| 3 | 5 | 28 | No |
| 4 | 7 | 28 | No |
| 5 | 3 | 28 | No |
| 6 | 13 | 28 | No |
| 7 | 22 | 28 | No |
| 8 | 6 | 28 | No |
| 9 | 18 | 28 | No |
| 10 | 5 | 28 | No |
| 11 | 3 | 28 | No |
| 12 | 5 | 5 | No |
| 13 | 5 | 28 | No |
| 14 | N/A | 11 | Yes  (perforation, 11th day) |
| 15 | 5 | 28 | No |
| 16 | 4 | 28 | No |
| 17 | 7 | 28 | No |
| 18 | 10 | 28 | No |
| 19 | N/A | 4 | Yes (antifungals, 4th day) |
| 20 | N/A | 112 | Yes  (big infiltrate, 1st day) |
| 21 | 3 | 14 | No |
| 22 | 5 | 28 | No |
| 23 | 28 | 28 | No |
| 24 | 7 | 28 | No |
| 25 | 9 | 28 | No |
| 26 | 14 | 28 | No |
| 27 | 3 | 5 | No |
| 28 | 5 | 10 | No |
| 29 | 12 | 16 | No |
| 30 | 10 | 28 | No |
| 31 | 18 | 27 | No |
| 32 | 34 | 44 | No |
| 33 | n/a | 69 | Yes (no healing) |
| 34 | 50 | 61 | No |
| 35 | 49 | 54 | No |
| 36 | 23 | 30 | No |
| 37 | 10 | 16 | No |
| 38 | 5 | 18 | No |
| 39 | 10 | 17 | No |
